# Treatment of Trigeminal Neuralgia (Tic Douloureux), with Report of a Case Successfully Treated with Nitroglycerin

**Published:** 1897-10

**Authors:** William C. Krauss

**Affiliations:** Buffalo, N. Y., Professor of nervous diseases, Medical Department of Niagara University


### TREATMENT OF TRIGEMINAL NEURALGIA (TIC DOULOUREUX), WITH REPORT OF A CASE SUCCESSFULLY TREATED WITH NITROGLYCERIN.

By WILLIAM C. KRAUSS, M. D., Buffalo, N. Y.,
Professor of nervous diseases, Medical Department of Niagara University.

AMONG the various forms of pain none baffle the skill and patience of the physician more than neuralgia of the fifth or trigeminal nerve, commonly called tic douloureux. So obstinate has this affection been that surgical intervention has, in many cases, been resorted to with a hope of allaying and stilling the excruciating paroxysms of pain. Neurectomy for many years was performed with varying success, but generally unfavorably, until the entire removal or destruction of the Gasserian ganglion was proposed and, in recent years, successfully carried out. Stretching of the nerve by Hueter's method was also in vogue at one time, but the beneficial results were only temporary and in a short time the usual paroxysms reappeared. Of the surgical procedures this last one was the simplest and the results equally so, while the neurectomy of the branches of the trigeminus, at their point of exit, gave a longer respite from pain and in many cases complete recovery followed. Thus, Tansini[1] reports a case of severe trigeminal neuralgia, where neurectomy of the nerve was performed at the supraorbital, infraorbital and mental foramina, followed by the application of the actual cautery to the central stump, which was completely successful. In two other cases the same procedure was also followed with success.

Transection of the nerve is not sufficient in these cases, as regeneration of the nerve elements rapidly takes place and with it the recurrence of the paroxysms. Either a considerable portion of the nerve must be excised or the central end of the nerve treated with the actual cautery. But even if these precautions have been taken it is not safe to assert that the paroxysms will not reappear. Gilles de la Tourette, from an abundance of material at the Salpêtrière, Paris, claims that in this disease neurotomy never gives lasting success. Dana[2] likewise draws attention to this procedure as a failure in affording permanent relief.

The latest and most difficult procedure is the removal or breaking up of the Gasserian ganglion. The difficulties met with, the severity of the operation and the results are very well illustrated by the summaries of Keen's cases,[3] which were operated upon under the most auspicious surroundings, as regards asepsis, method and operator.

CASE I.—Breaking up of the Gasserian ganglion after thirteen prior operations. Done in two stages on account of packing to arrest the hemorrhage; recovery; cure for twenty-six months, possible slight return. (?)

CASE II.—Breaking up of the Gasserian ganglion, in two stages, after eight prior operations. Recovery; cure for eighteen months; possible slight return.

CASE III.—Breaking up of the Gasserian ganglion after two prior operations; rupture of the middle meningeal at the foramen spinosum; infection during operation; death from septic meningitis.

CASE IV.—Breaking up of the Gasserian ganglion after five prior operations; done in two stages on account of hemorrhage; packing with gauze for three days; recovery; cure for seven months; necrosis of the bone in the flap.

CASE V.—Curetting of the Gasserian ganglion after two prior operations. Rupture of the middle meningeal at the foramen spinosum. Post-operative corneal ulcer; recovery; cure for two months.

CASE VI.—Removal of the entire Gasserian ganglion with its second and third sections and its sensory and motor seats back to the pons in a piece four centimeters long, after four prior operations; recovery; cure for three weeks; post-operative corneal ulcer.

Keen operated by the Krause-Hartley method, which is preferred over the Rose method on account of lesser mortality, more ready access to the ganglion and the facility with which it can be removed. In twenty-two cases done by the Rose method the mortality has been four, or eighteen per cent., while in fifty-one operations, done by the Krause-Hartley method, the mortality has been only five, or 9.8 per cent. Keen states that so far as he knows there have been only three recurrences after operation, one in Rose's practice and two in his own. If this is a true index of the results of the operation, it recompenses for all the trials and dangers that patient and operator pass through. The resection of Meckel's ganglion has also been done, especially by Guinard,[4] who reported three cases which had been free from pain for over two years.

No mention need be made here of those cases of paroxysms of the third division, due to disease of the antrum of Highmore or to carious teeth, as they generally improve rapidly after the irritating conditions are removed.

From a purely medical standpoint tic douloureux has conquered the *vis medicatrix* almost completely—only here and there a case where some form of treatment has been of any benefit. What has been of service in one case has been useless in another, and in the pure neuralgic (not irritative) condition, a score or more of drugs have been recommended for trial. The most attention has been paid to aconitine, but as the preparations of this drug are unstable, and therefore unreliable, a difficulty is at once encountered. Stillé, in his fourth edition, in speaking of neuralgia said that "in the treatment of this distressing affliction the benefits to be derived from aconite are unequivocal and precious." At the 1896 meeting of the Association of American Physicians, in discussing the treatment of trigeminal neuralgia, Janeway said it was very difficult to get reliable aconitine, but when a good article was used the results were often very good. Putnam, of Boston, said he had been moderately successful with aconitine, but was willing to try the strychnia treatment as recommended by Dana. Hunsberger[5] reported in a paper before the Pennsylvania State Society, 1895, his results with aconitine in fifty-seven cases of neuralgia of various kinds and thinks it superior to any other remedy, even operation. One of these cases was neuralgia of the fifth nerve, in which he administered "1-200 grain of aconitine every two hours with no apparent effect until the second day, when the pains gradually subsided, and in a week's time, or rather six days' time, she was freer from pain than she had been for fifteen years. I continued the aconitine for two months. She has had paroxysms of pain at intervals since, but the exhibition of aconitine for a few days would always quiet the nerve storm."

In an editorial on the value of aconitine in facial neuralgia in *Therapeutic Gazette*, July 15, 1896, is mentioned the fact—which in all probability is overlooked by many physicians—that there are two forms of aconitine upon the market, one of which is *amorphous*, the other *crystalline.* The latter possesses greater usefulness in neuralgic conditions, and that known as Duquesnel's should always be preferred.

In speaking of the dosage, the editorial asserts that because of the uncertainty and impurity of many of the specimens which have been tried by many physicians, the dose of pure aconitine is as yet undecided. Thus it is stated that as much as two and one-half grains of so-called aconitine has been taken with impunity, and that on the other hand a fatal dose of poisoning has resulted from the administration of 1-128 grain. Furthermore than this, the United States Dispensatory asserts that 1-640 grain is probably the largest amount that ought to be administered. On the other hand it is commonly stated by a number of authors that the dose is 1-100 grain, while Dr. Hunsberger gave 1-200 grain every two hours.

The editorial concludes as follows:

> The general trend of professional opinion seems to be that owing to the powerful, poisonous influence of this alkaloid and the uncertainty of its dose, it is not justifiable to administer it internally. Personally, we would feel that if we did give it internally we would be running a considerable risk and, in the event of an accident, would be held responsible for any disagreeable results which might ensue.

If such is the judgment upon the use of aconitine, it is time that the use of the drug should be curtailed and given only in extreme cases and as a last resort.

Dana has recommended the hypodermatic injection of strychnia; the injections are made once a day and the dose gradually increased from 1-30 grain to 1-5 grain or ¼ grain, ten to twenty days being required to reach this maximum. He says the large doses sometimes have an immediate anodyne effect like that of morphia. Six cases were reported, two in women, four in men, and all were relieved but one. The period of relief in the five cases had been from one and one-half to two years.

The injection of cocaine into the arm has been advocated by Seagrave,[6] the effect being not so much local as general, probably through the vaso-motor influence. Gilles de la Tourette advises the administration of thebaic extract. The first day he gives two pills, each containing two centigrammes of the drug. The quantity is gradually increased until doses of twenty centigrammes are reached, and then gradually decreased to two centigrammes and then stopped entirely.

Local external applications have been of some service, especially the oleate of aconitine, which must be employed very carefully; also compresses wet with from fifteen drops to one dram of guiacol[7] applied over the painful spots for a very short time.

Quinine, iron, arsenic, and the fats, also the Brown-Sèquard shotgun pill, have all, in some cases, rendered fair service, but aside from their tonic virtues probably do not exert much influence as anti-neuralgics. They do form important adjuncts to treatment of whatever nature and must not be relegated to the rear as obsolete and antiquated.

Some of the synthetical compounds and the opiates are of great service in temporarily checking the pain, but their use is to be held in check as much as possible.

Having succeeded admirably with nitroglycerine in cases of sciatic neuralgia,[8] I determined to try this drug in tic douloureux and watch its effect in relieving pain.

I was called in consultation, in November, 1896, with Drs. Mynter and Redmond to see one of the cloistered nuns on Best street, who had been having for some time past an inveterate trigeminal neuralgia. It was thought probable that an operation would be more liable to give the desired relief, and the removal of the Gasserian ganglion was contemplated. The surgeon not wishing to operate until all medicinal agents had been carefully employed, delayed, until, at my suggestion, nitroglycerine had been thoroughly tested. I prescribed tablets of 1-100 grain of the drug, at first three times daily and increasing until she was taking seven or eight tablets daily and if necessary one tablet every hour. The face was also treated with the mild galvanic current and a laxative tonic to improve her general condition. She began to improve after the second week and has steadily continued without interruption. The large doses of nitroglycerine did not affect her disagreeably and the head did not pulsate as is sometimes noted.

Tolerance to nitroglycerine is easily acquired by some patients. Thus Stewart[9] reports a case of chronic nephritis where six months after the initial dose, the patient was taking fifty minims of a ten per cent. solution (five minims of pure nitroglycerine), four times daily, with less effect on the vascular tension than the initial dose of 1-100 grain.

Reading[10] narrates the case of a woman, 57 years old, suffering with chronic interstitial nephritis, who, less than one year after the initial dose of nitroglycerine, was taking one teaspoonful of the ten per cent. solution at each dose, this being equal to six grains of the pure drug.

The dose of my patient compared to these two is insignificant, and the moral to be drawn is to increase the dose until the physiological effects are produced, even if enormous doses must be administered.

Aside from its anti-neuralgic qualities it is a tonic, and it is reported that girls working in nitroglycerine factories become plump and rosy and are the pictures of health.

1. British Medical Journal, March 16, 1895.
2. Medical Record, May 9, 1896.
3. American Journal of the Medical Sciences, January, 1896.
4. Gazette des Hôpitaux, 1895, p. 1251.
5. Therapeutic Gazette, August 15, 1895, p. 510.
6. British Medical Journal, February 8, 1896.
7. Ferrand, Ind. Med. Chir. Rev., March, 1896.
8. New York Medical Journal' February 29, 1896.
9. Therapeutic Gazette, September 15, 1893.
10. Therapeutic Gazette, May 15, 1893, p. 292.

371 Delaware Avenue.

---

Dr. Willis F. Westmoreland, professor of surgery in the Atlanta Medical College, is authority for the statement that Dr. Warren's declaration (in his text-book of Surgical pathology, 1895,) that "carcinoma does not occur in the negro" is wholly incorrect. He has seen at least a half dozen cases in the last two years. There can, however, be no question that the disease is far less frequent in the African than in the Caucasian race.—*Am. Jour. Surg. and Gynec.*

